# Supplementary material for: Uncertainty reduction in biochemical kinetic models: Enforcing desired model properties
Source: PLoS Comput Biol. 2019 Aug 20;15(8):e1007242. doi: 10.1371/journal.pcbi.1007242 (PMC6716680; doi:10.1371/journal.pcbi.1007242)
Supplement: S1 Text — (DOCX) [file pcbi.1007242.s003.docx]

## List of abbreviations

#### Enzymes

*XTR*, hexose transporters for xylose; *XRI* and *XRII*, xylose reductase; *XLT*, xylitol excretion transporter; *XDH*, xylitol reductase; *XK*, xylulokinase; *GTR*, hexose transporters for glucose; *HXK*, hexokinase; *PGI*, glucose-6-phosphate isomerase; *PFK*, phosphofructokinase; *FBA*, fructose-biphosphate aldolase; *TPI*, triose phosphate isomerase; *TDH*, glyceraldehyde-3-phosphate dehydrogenase; *PGK*, phosphoglycerate kinase; *GPM*, phosphoglycerate mutase; *ENO*, enolase; *PYK*, pyruvate kinase; *ZWF*, glucose-6-phosphate-1-dehydrogenase; *RKI*, ribose-5-phosphate isomerase; *RPE*, ribulose-5-phosphate 3-epimerase; *TKL1*, transketolase; *TKL2*, transketolase; *TAL*, transaldolase; *PDC*, pyruvate decarboxylase; *ALD*, aldehyde dehydrogenase; *ACS*, Acetyl-CoA synthase; *CAT*, carnitine o-acetyltransferase; *ACARt*, acetylcarnitine diffusion; *YAT*, carnitine o-acetyltransferase; *CARt*, carnitine diffusion; *PYRt*, pyruvate carrier; *PDA*, pyruvate dehydrogenase; *PYC*, pyruvate carboxylase; *PCK*, phosphoenolpyruvate carboxylkinase; *OAtrans*, oxaloacetate carrier; *MAE*, malic enzyme; *CIT*, citrate synthase; *ACO*, aconitase; *IDH*, isocitrate dehydrogenase; *KGD*, a-ketoglutarate dehydrogenase; *LSC*, succinate-CoA ligase; *SDH*, succinate dehydrogenase; *FUM*, fumarase; *MDH*, malate dehydrogenase; *NDH*, external NADH dehydrogenase; *NDI*, NADH dehydrogenase; *FDH*, FADH2 dehydrogenase; *NDR*, NADPH reductase; *QCR*, ubiquinol cytochrome C reductase; *COX*, cytochrome C oxidase; *ASN*, ATP synthase; *AAC*, ADP/ATP carrier protein; *ADK*, adenylates kinase; *ATPmt*, ATP maintenance; *ADH*, cytosolic alcohol dehydrogenase; *SCD,* succinate dehydrogenase (ubiquinone-6), mitochondrial; *ACET*, acetate diffusion; *COH*, carbonic acid hydro-lyase; *PPP,* Pyrophosphate phosphohydrolase; *MLPIT,* malate transport, mitochondrial; *ICL,* Isocitrate glyoxylate-lyase; *MLS,* L-Malate glyoxylate-lyase (CoA-acetylating); *MDHc,* (S)-malate:NAD+ oxidoreductase; *CITc,* Citrate oxaloacetate-lyase cytosolic; *ACOc*, citrate hydro-lyase cytosolic; *LACm2r,* D-lactate transport, mitochondrial; *CITt2m,* citrate transport, mitochondrial; *LDH,* (R)-Lactate:ferricytochrome-c 2-oxidoreductase; *O2m,* O2 transport (diffusion); *CO2m,* CO2 transport (diffusion), mitochondrial; *PIm,* phosphate transporter, mitochondrial; *CO2t,* CO2 transport via diffusion; *GLYCt,* glycerol transport in/out via diffusion reversible; *PYRst,* Pyruvate transport via proton symport; *SO4t,* sulfate transport via proton symport; *Pit,* phosphate transport via proton symport; *O2t,* O2 transport via diffusion; *NH4t,* Ammonia transport via diffusion; *LACt2r,* D-lactate transport via proton symport; *SUCCt2r,* succinate transporter in/out via proton symport; *MALt2r,* L-malate transport in via proton symport; *GND1,* 6-Phospho-D-glucono-1,5-lactone lactonohydrolase; *GND2,* 6-Phospho-D-gluconate:NADP+ 2-oxidoreductase (decarboxylating); *GPD1,* Glycerol-3-phosphate:NAD+ 2-oxidoreductase; *GPD2,* Glycerol-3-phosphate phosphohydrolase.

#### Chemical species

XL, xylose; XLT, xylitol; XYLL, xylulose; GLC, glucose; G6P, glucose-6-phosphate; F6P, fructose-6-phosphate; FBP, fructose 1,6-diphosphate; T3P, glyceraldehydes-3-phosphate; DHAP, glycerone phosphate; DPG, bisphosphoglycerate; 3PG, 3-phosphoglycerate; 2PG, 2-phosphoglycerate; PEP, phosphoenolpyruvate; PYR, pyruvate; 6PGL, glucono-1,5-lactone 6-phosphate; RL5P, ribulose 5-phosphate; R5P, ribose 5-phosphate; X5P, xylose-5-phosphate; E4P, erythrose 4-phosphate; S7P, sedoheptulose 7-phosphate; AALD, acetaldehyde; ACET, acetate; ACCOA, acetyl-CoA; CAR, carnitine; ACAR, acetylcarnitine; OAA, oxaloacetate; CIT, citrate; ICIT, isocitrate; AKG, 2-oxoglutarate; SUCCOA, succinyl-CoA; SUCC, succinate; FUM, fumarate; MAL, malate; GL, glycerol; ETH, ethanol; CO2; HCO3-; O2; PPi, Pyrophosphate; Pi, Phosphate; NH4; SO4; 6PGC, 6-Phospho-D-gluconate; GLYC3P, glycerol-3-phosphate; ACET, acetate; LAC, D-lactate; GLYX, glyoxylate.
