# Supplementary figures and images for: Uncertainty reduction in biochemical kinetic models: Enforcing desired model properties
Source: PLoS Comput Biol. 2019 Aug 20;15(8):e1007242. doi: 10.1371/journal.pcbi.1007242 (PMC6716680; doi:10.1371/journal.pcbi.1007242)

**Boxes Size/FI distribution depending on classification method (30 boxes)**

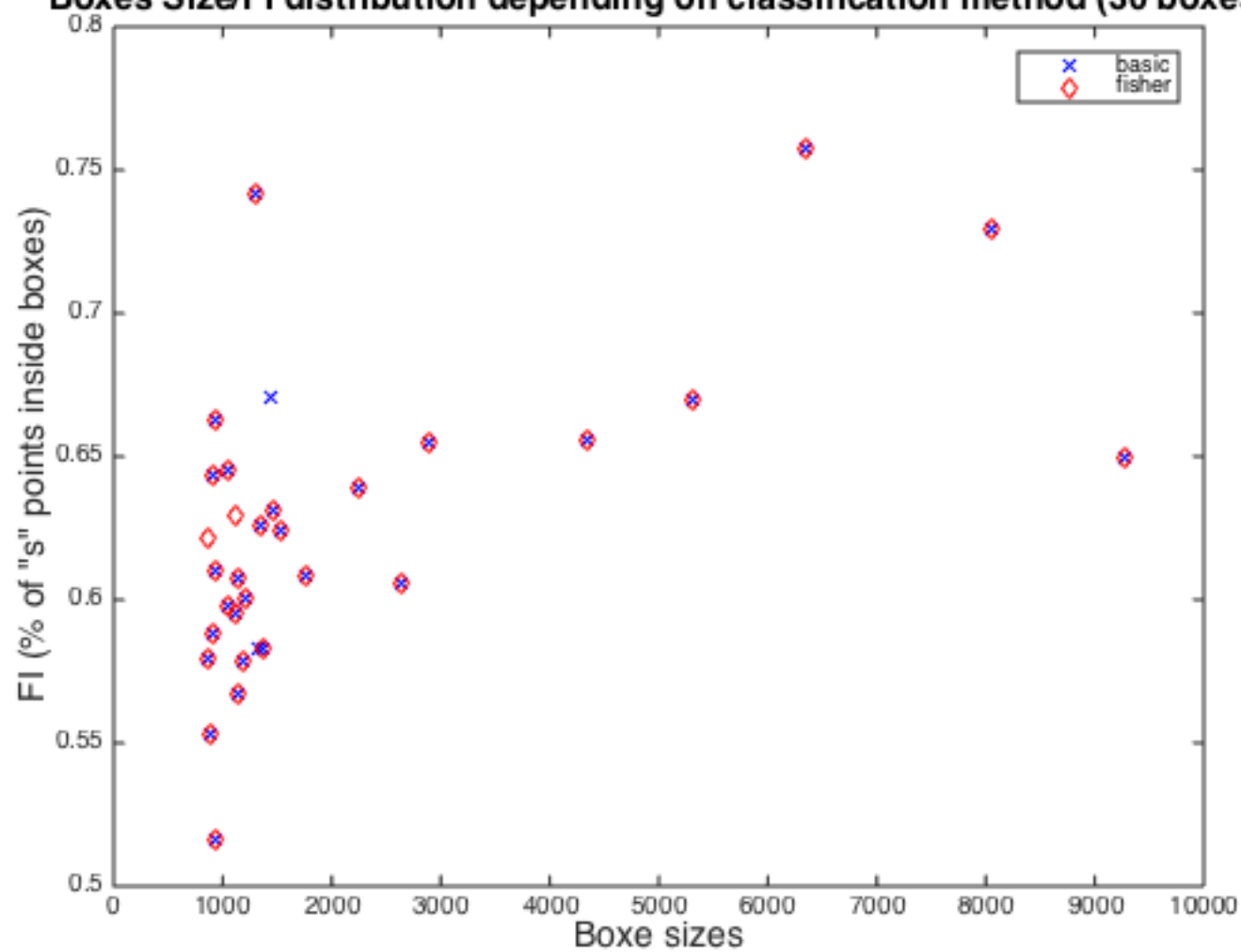

Supplement: S1 Fig — The rules from a tree training with all parameters (blue crosses), and the rules from a tree training with a reduced set of parameters (red diamonds) coincide in the majority of instances. (PDF) [file pcbi.1007242.s007.pdf]
